# Change of Circulating and Tissue-Based miR-20a in Human Cancers and Associated Prognostic Implication: A Systematic Review and Meta-Analysis

**DOI:** 10.1155/2018/6124927

**Published:** 2018-11-25

**Authors:** Qingyu Zhang, Qiwei Wang, Wei Sun, Fuqiang Gao, Lihua Liu, Liming Cheng, Zirong Li

**Affiliations:** ^1^Graduate School of Peking Union Medical College, China-Japan Friendship Institute of Clinical Medicine, Chaoyang District, Beijing 100029, China; ^2^Department of Orthopedics, Peking University China-Japan Friendship School of Clinical Medicine, Beijing 100029, China; ^3^Centre for Osteonecrosis and Joint-Preserving & Reconstruction, Orthopaedic Department, China-Japan Friendship Hospital, Graduate School of Peking Union Medical College, Chaoyang District, Beijing, China; ^4^Centre for Osteonecrosis and Joint-Preserving & Reconstruction, Orthopaedic Department, China-Japan Friendship Hospital, Chaoyang District, Beijing, China

## Abstract

**Background:**

Previous literatures have investigated the change of miR-20a expression level in the progression of multiple cancers and its influence on patients' survival outcome, but results of now-available evidence are inconsistent.

**Objective:**

To elucidate the prognostic value of circulating and tissue-based miR-20a for patients with various cancers.

**Methods:**

A systematic search and review of eligible publications were carried out in three electronic databases including the Cochrane Library, PubMed, and Embase, and the methodological quality of included studies was assessed according to Newcastle-Ottawa Scale (NOS). Hazard ratios (HRs) and corresponding 95% confidence intervals (CIs) for overall survival (OS), recurrence-free survival (RFS), disease-free survival (DFS), and progressive-free survival (PFS) of each study were pooled using a random effect model.

**Results:**

In total, 24 studies involving 4186 samples of multiple cancers published in 20 articles were included in the statistical analysis. As for circulating miR-20a, five kinds of cancers containing gastric cancer, lymphoma, glioblastoma, prostate cancer, and non-small-cell lung cancer reported upregulated level in patients compared with normal healthy control, and overexpressed circulating miR-20a could confer an unfavorable factor for OS (HR = 1.71, 95% CIs: 1.43 -2.04,* p* < 0.01) and DFS (HR = 1.90, 95% CIs: 1.45-2.49,* p* < 0.01). As for tissue-based samples, 6 kinds of malignancies including colorectal cancer, salivary adenoid cystic carcinoma, gallbladder carcinoma, colon cancer, gastrointestinal cancer, and alveolar rhabdomyosarcoma revealed upregulated miR-20a expression level compared with paired nontumorous tissue, of which high expression of miR-20a was significantly associated with poor OS (HR = 2.74, 95% CIs: 1.38-5.42,* p* < 0.01) and DFS (HR = 2.68, 95% CIs: 1.32-5.45,* p* < 0.01); meanwhile, other 5 tumors containing breast cancer, cutaneous squamous cell carcinoma, hepatocellular carcinoma, oral squamous cell carcinoma, and epithelial ovarian cancer demonstrated downregulated miR-20a expression level compared with benign tissue, of which low miR-20a expression was significantly related to shorter OS (HR = 3.48, 95% CIs: 2.00-6.06,* p* < 0.01) and PFS/RFS (HR = 4.05, 95% CIs: 2.89-5.66,* p* < 0.01).

**Conclusion:**

Change of circulating and tissue-based miR-20a expression possesses vital prognostic implication for human cancers. Augmented expression of circulating miR-20a predicts poor survival outcome for patients with cancers. Tissue-based miR-20a level may be upregulated or downregulated depending on cancer types; in the former condition, high expression of tissue miR-20a is a risk factor for unfavorable prognosis and in the latter condition low expression of tissue miR-20a is associated with shorter survival.

## 1. Introduction

MicroRNAs (miRNAs/miRs) are a set of single-stranded, nonprotein-coding RNAs approximately 19~24 nucleotides in length [1]. It is demonstrated that miRNAs are highly conservative in evolution and act as posttranslational inhibitors by binding to the complementary sequences in the 3′ untranslated regions (3′-UTR) of messenger RNAs (mRNAs) and therefore leading to translation regression or triggering decay factors of mRNAs [1, 2]. Due to the fact that majority of encoding sequences of miRNAs lie in cancer-related regions of genome [3], dysregulated miRNAs profile often plays a profound role in various tumor-associated biological processes such as proliferation, differentiation, migration, angiogenesis, stress response, metabolism, invasion, chemoresistance, and apoptosis [1, 3]. In these years, numerous miRNAs have emerged as candidates of molecular biomarkers for diagnosing human cancers and guiding treatment as well as predicting the metastasis, relapse, and prognosis [3–5].

MiR-20a is a typical and extensively investigated example of miRNAs originating from the miR-17~92 cluster, which is located at chromosomal locus 13q31.3 and able to encode five other mature miRNAs including miR-17, miR-18a, miR-19a/b, and miR-92a [6]. MiR-20a is identified in a wide range of clinical specimens (plasma, serum, tissue, feces, etc.) and the expression pattern of circulating and tissue-based miR-20a can characterize multiple human cancers [7, 8]. Zhang and his colleagues demonstrated that miR-20a level in cutaneous squamous cell carcinoma had a close relationship with tumor stage [9]; Sanfiorenzo et al. proposed that a six-miRNA plasma panel comprising miR-20a, miR-145, miR-24, miR-152, miR-25, and miR-199a was able to discriminate non-small-cell lung cancer (NSCLC) from chronic obstructive pulmonary disease (COPD) and indicate recurrence in resectable NSCLC [10]. Accumulating studies attempted to testify the clinical impact of the expression pattern of circulating and tissue-based miR-20a for human cancers, but their study designs were various, and results were inconsistent [11–18]. MiR-20a expression level is downregulated in several kinds of malignancy while upregulated in others; meanwhile, some studies exploited the expression level of miR-20a in serum or plasma, and some in tumorous and nontumorous tissues [7].

Although six mature miRNAs could be encoded by miR-17~92 cluster, the diverse sequence of each miRNA results in their specificity of target genes and separate physiobiological functions [17]. Moreover, studies appraising diagnostic significance of multiple miRNAs were often based on same group of population [13, 16–18]. Therefore, indiscriminately pooling data of all these miRNAs is inappropriate. On the other hands, if we make separative quantitative appraising of miRNAs related to miR-20a, these workloads are heavy, and the theme of this article will become ambiguous. Therefore, in this article, we only chose miR-20a, a widely investigated miRNA with controversy prognostic value in human cancers, as the target of interest.

Two previous meta-analyses with respect to the prognostic value of miR-17~92 cluster in various tumors were published in 2017[19, 20]. These analyses demonstrated that high expression of miR-17~92 cluster was significantly predictive of a poor prognosis in various cancers, with pooled risk ratios of 2.05 (95% confidence interval (CI) 1.58-2.65) and 1.71 (95% CI 1.39-2.11) for overall survival (OS), respectively. But these two meta-analyses only included 6 and 7 studies appraising prognostic value of miR-20a in cancer patients and evaluated all members of miR-17~92 cluster as a whole. Moreover, investigators did not distinguish different sources of tested samples (circulating or tissue-based) or perform subgroup analysis according to the trend of miR-20a change. Recently, an increasing amount of studies about circulating or tissue-based miR-20a have been published [9–11, 13–15, 17–20]. Therefore, we performed this updated systematic review and meta-analysis to authentically and comprehensively assess the value of miR-20a for monitoring therapeutic efficacy and prognosis of human cancers.

## 2. Materials and Methods

We followed the preferred reporting items for systematic reviews and meta-analyses (PRISMA) statement in the conduction of this study [21]; each process was performed by two investigators (Qingyu Zhang and Qiwei Wang) repeatedly and independently, and any disagreement was resolved by discussion or arbitrating by a third author (Wei Sun). Informed consent was not requisite because all data were extracted from published articles.

### 2.1. Literature Searching and Including

Three electronic databases including PubMed, Embase, and the Cochrane Library were searched for eligible literatures using following keywords: microRNA-20a OR miRNA-20a OR miR-20a OR microRNA-20a-5p OR miRNA-20a-5p OR miR-20a-5p. Then the reference lists of relevant articles were also checked by hands to retrieve other potentially qualified publications. Last search was updated on May 1, 2018, and no language limitations were imposed.

Eligible studies for this meta-analysis had to confirm following criteria: (1) population, patients with any kind of cancer; (2) the association between miR-20a expression level and prognosis was assessed; (3) the primary outcome was overall survival (OS) or relapse-free survival (RFS) or progression-free survival (PFS) or disease-free survival (DFS); (4) enough data were provided to obtain trustworthy hazard ratios (HRs) and corresponding 95% confidence intervals (CIs); and (5) nonoriginal articles (case reports, reviews, letters, meta-analyses, and editorials), meeting abstracts, and animal studies were excluded. If there were studies with duplicate cohorts, only the more comprehensive or recent one was enrolled.

### 2.2. Data Extraction

For included studies, collected information was as follows: first authors' surname, year of publication, original country, sample size, demographic information of participants (age, year, and so on), type and stage of cancer, source of sample, duration of follow-up, method of detecting miR-20a expression, level of miR-20a, survival outcome, and cut-off values. For each study, HRs and associated 95% CIs were extracted directly if they were provided explicitly in original articles or supplementary materials (available here); otherwise, they were calculated by using log-rank/Cox regression statistics by Tierney's methods [22]. Sample source was categorized into tissue, serum, and plasma, while sample sizes were divided into those of more than 100 and those less than 100. HRs calculated from multivariate regression analysis model were preferred to adjust the confounders and if not provided, those from univariate analysis were extracted. These data were filled into a predesigned excel file for further analysis and calculation.

### 2.3. Quality Assessment

Newcastle-Ottawa Scale (NOS) was used to appraise the methodological quality of included studies. This tool compromises nine items and one score was earned if information concerning this item was clearly reported in original studies. Studies of ≥ 7 were regarded as high-quality reporting.

### 2.4. Statistical Methods

HRs and corresponding 95% confidence intervals (CIs) of individual study were pooled to obtain the summary estimates by using a random effect model (DerSimonian and Laird method). The heterogeneity across studies was assessed using the Cochran Q and I^2^ index. I^2^ of < 25% represents small heterogeneity, 50% ≥ I^2^ ≥ 25% moderate heterogeneity, and I^2^ > 50% significant heterogeneity. One-way sensitivity analyses were conducted by removing publications individually to evaluate the stability of results. Meanwhile, subgroup analyses based on region of publication, cancer type, sample size, and calculation model of HR (multivariate or univariate) were performed. We utilized Egger's test to detect any possible publication bias (significant publication bias if a two-tailed* p* value < 0.05).

All analyses were performed with STATA, version 12.0 (StataCorp, College Station, TX, USA).

## 3. Results

### 3.1. Literature Selection and Study Characteristics

**Figure 1:**
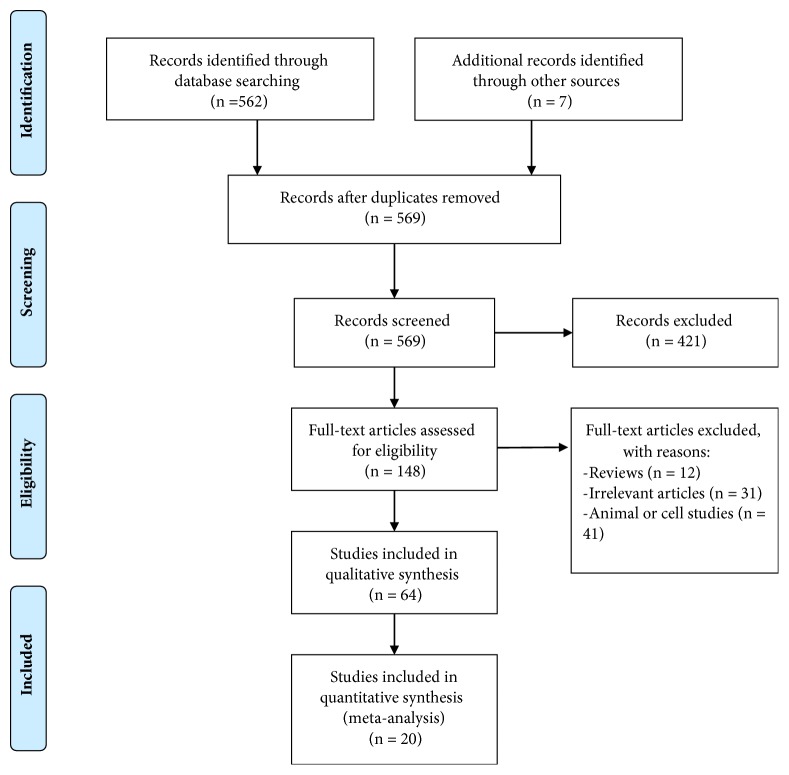
Flow diagram of literature research and selection process.

568 nonduplicated publications were retrieved by searching three databases and screening references of relevant articles, and eventually 20 [9–11, 13–15, 17, 18, 34] articles were included in statistical analysis. The literature search and selection processes were summarized in Figure 1 and the basic information of included studies was described in Tables 1 and 2. Among these studies, Chen and his colleagues validated prognostic impaction of miR-20a by analyzing colorectal cancer patients in Tumor Cancer Genome Atlas (TCGA) databases [26]; Zhang et al. randomly assigned patients to the training set, internal testing set, and independent validation set to testify the prognostic value of miR-20a in stage II~IV colon cancer [28]; meanwhile, Si et al. presented data about association between miR-20a and breast cancer patients' survival in three different cohorts (cohorts 1 and 2 from their own research center and cohort 3 from TCGA databases) [32]. These datasets from the same articles were collected simultaneously, and eventually this meta-analysis was established based on 24 studies.

All 20 literatures were written in English. As for methodological quality of 24 included studies, four [10, 14, 17, 33] got 8 score and fifteen [9, 11, 18, 23, 25, 26, 28, 31, 32, 32, 34] studies had 7 score. Only five [13, 15, 24, 29, 30] studies received 6 score. Mean NOS score for these studies was 6.96 (range 6~8). The sample sizes ranged from 25 to 716 with a total of 4186 samples and 16 kinds of tumors were analyzed: gastric cancer, lymphoma, glioblastoma, prostate cancer, NSCLC, colorectal cancer, salivary adenoid cystic carcinoma, gallbladder carcinoma, colon cancer, gastrointestinal cancer, alveolar rhabdomyosarcoma, breast cancer, cutaneous squamous cell carcinoma, hepatocellular carcinoma, oral squamous cell carcinoma, and epithelial ovarian cancer. 11 types of tumors whose sample source was cancerous and noncancerous tissue were reported in 17 [14, 15, 17, 18, 26–30, 33, 34] studies (2 colorectal cancer, 1 salivary adenoid cystic carcinoma, 1 gallbladder carcinoma, 4 colon cancer, 1 gastrointestinal cancer, 1 alveolar rhabdomyosarcoma, 3 breast cancer, 1 cutaneous squamous cell carcinoma, 1 hepatocellular carcinoma, 1 oral squamous cell carcinoma, and 1 epithelial ovarian cancer); meanwhile, 7 [9–11, 13, 24, 25] studies testing circulating miR-20a expression involved a total of 5 kinds of tumors (2 gastric cancer, 1 lymphoma, 1 glioblastoma, 1 prostate cancer, and 1 NSCLC). All studies used real-time polymerase chain reaction (RT-PCR) to test the targeted miRNA. The cut-off value for high and low expression of miR-20a was various in included studies. As for the survival outcomes, 24 eligible studies could be divided into 30 datasets: 20 for OS, 7 for DFS, 2 for PFS, and 1 for RFS (see Table 2).

### 3.2. Circulating miR-20a and Cancer Prognosis

All 6 kinds of tumors involving circulating miR-20a shown upregulated miR-20a expression level in patients compared with the healthy control [9–11, 13, 25].

#### 3.2.1. Circulating miR-20a and Overall Survival

**Figure 2:**
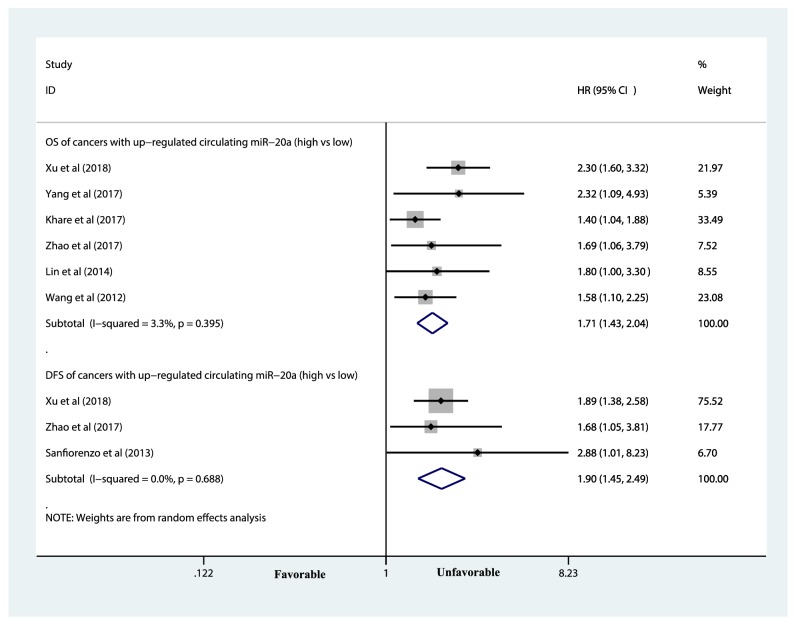
Forest plot of the association between high expression of circulating miR-20a and survival in various cancers. The area of the square represented the weight of each study in the pooled results. The diamond indicated pooled HR and corresponding 95% CI. As depicted, the diamond in the right of the central vertical line represents an unfavorable prognosis in the former group in comparison with the latter group. CI, confidence interval; HR, hazard ratio.

5 [9, 11, 13, 24, 25] studies provided HRs and corresponding 95% CIs for the association between circulating miR-20a expression and overall survival. Among those tumors, high expression of circulating miR-20a was significantly associated with unfavorable OS of cancer patients (HR = 1.71, 95% CIs: 1.43 -2.04,* p* < 0.01;* p* for heterogeneity = 0.395, I-square = 3.3%) (see Figure 2 and Table 3).

#### 3.2.2. Circulating miR-20a and Disease-Free Survival

3 [10, 23, 25] studies provided HRs and corresponding 95% CIs for DFS, and high expression of miR-20a was associated with unfavorable pooled DFS (HR = 1.90, 95% CIs: 1.45-2.49,* p* < 0.01;* p* for heterogeneity = 0. 688, I-square = 0.0%), too (see Figure 2 and Table 3).

### 3.3. Tissue-Based miR-20a and Cancer Prognosis

6 kinds of tumors (colorectal cancer, salivary adenoid cystic carcinoma, gallbladder carcinoma, colon cancer, gastrointestinal cancer, and alveolar rhabdomyosarcoma) show upregulated miR-20a expression level in tumorous tissue compared with nontumorous tissue and other 5 cancers (breast cancer, cutaneous squamous cell carcinoma, hepatocellular carcinoma, oral squamous cell carcinoma, and epithelial ovarian cancer) demonstrated downregulated miR-20a expression level in tumorous tissue compared with paired nontumorous tissue.

#### 3.3.1. Tissue miR-20a and Overall Survival

**Figure 3:**
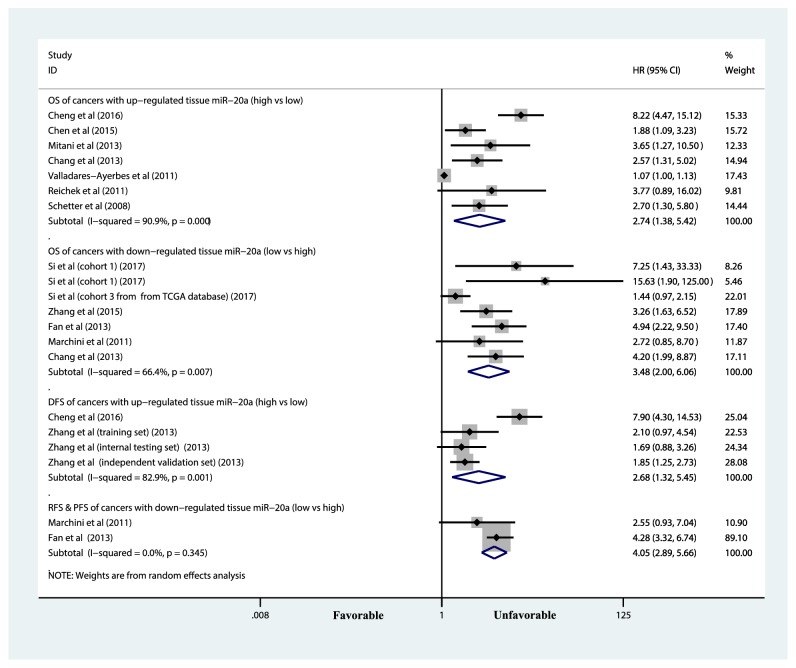
Forest plot of the association between tissue-based miR-20a and survival in various cancers. The area of the square represented the weight of each study in the pooled results. The diamond indicated pooled HR and corresponding 95% CI. As depicted, the diamond in the right of the central vertical line represents an unfavorable prognosis in the former group in comparison with the latter group. CI, confidence interval; HR, hazard ratio.

There were 14 [14, 15, 17, 18, 26, 27, 29, 30, 34] studies which reported HRs and corresponding 95% CI for the correlation between OS and expression of tissue-based miR-20a. Among those tumors with upregulated miR-20a level, highly expressed miR-20a was negatively associated with OS (HR = 2.74, 95% CIs: 1.38-5.42, p < 0. 01;* p* for heterogeneity < 0. 001, I-square = 90.9%) (see Figure 3 and Table 3). Meanwhile, among those tumors with downregulated miR-20a level, low expression of miR-20a notably indicated reduced OS (HR = 3.48, 95% CIs: 2.00-6.06, p < 0. 01;* p* for heterogeneity = 0. 007, I-square = 66.4%) (see Figure 3 and Table 4).

#### 3.3.2. Tissue-Based miR-20a and Disease-Free Survival

4 [14, 28] studies investigated impact of tissue-based miR-20a on DFS of cancer (Zhang et al.'s article contains three independent studies). Among those tumors with upregulated miR-20a level, highly expressed miR-20a was associated with unfavorable OS (HR = 2.68, 95% CIs: 1.32-5.45, p < 0. 01;* p* for heterogeneity = 0. 001, I-square = 82.9%) (see Figure 3 and Table 3).

#### 3.3.3. Tissue-Based miR-20a and Relapse-Free/Progression-Free Survival

2 [33, 34] studies provided HRs and corresponding 95% CI for the relationship between RFS/PFS of cancer patients and tissue-based miR-20a expression. Among those tumors with upregulated miR-20a level, low expression of miR-20a was significantly associated with unfavorable RFS/PFS (HR = 4.05, 95% CIs: 2.89-5.66, p < 0. 01;* p* for heterogeneity = 0. 345, I-square = 0.0%) (see Figure 3 and Table 4).

### 3.4. Subgroup Analysis and Sensitivity Analysis

**Figure 4:**
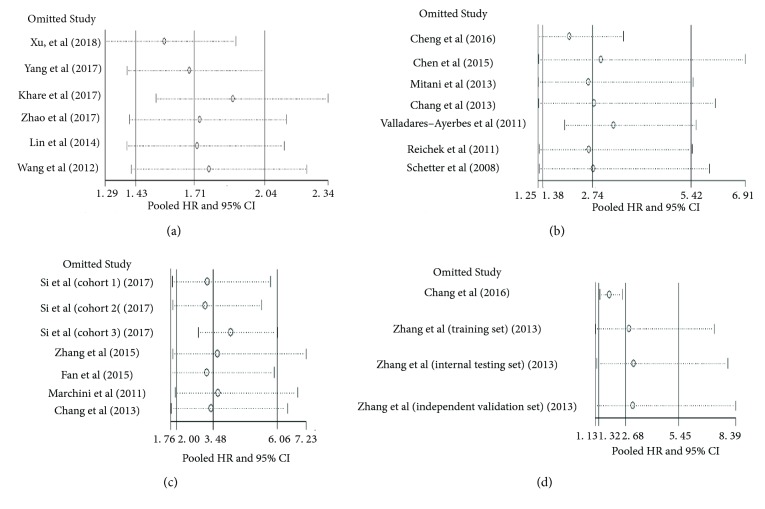
One-way sensitivity analyses about the association between miR-20a expression and survival in various cancers. (a) high expression of circulating miR-20a and OS; (b) tissue-based miR-20a and OS in cancers with upregulated miR-20a expression (high versus low); (c) tissue-based miR-20a and OS in cancers with downregulated miR-20a expression (low versus high); (b) tissue-based miR-20a and DFS in cancers with upregulated miR-20a expression (high versus low). Sensitivity analyses demonstrated that results of these meta-analyses were stable when omitting one study in each turn.

Above results were stable in the one-way sensitivity analysis when omitting studies one by one (see Figure 4). Meanwhile, subgroup analyses were conducted according to several factors such as region of publication (China or other countries), cancer type (gastrointestinal cancers or other cancers), sample size (< or > 100 participants), and calculation model of HR (multivariate or univariate), and the results demonstrated that these factors did not significantly change the relations between miR-20a and cancer prognosis (see Tables 3 and 4**)**.

## 4. Discussion

Identification of validated risk factors of various cancers could not only lead to a better understanding of molecular signaling pathways in the pathogenesis and progression of corresponding diseases but also offer new targets for clinical diagnosis and treatment [35]. Among alternative prognostic biomarkers such as plentiful proteins, RNAs, and DNAs, miRNAs seem to hold great promise and multiple profiling techniques (miRNA microarray, qRT-PCR, and next-generation sequencing) have been used for the measurement of circulating and tissue-based miRNAs [7]. MiRNA dysregulations in human cancer are mainly due to genomic changes including amplification, mutation, deletion, and disturbance of miRNA biogenesis enzymes, namely, Drosha, exportin 5, Dicer, and argonaute 2 (ARO2), and effects of these changes show cell/organ specificity [36–38].

Aberrant miR-20a level was observed in the progression of multiple human cancers but its efficacy as a prognostic biomarker remains inconsistent. In fact, a line of evidence has indicated that miR-20a could regulate a series of genes, where the effects may be either oncogenic or tumor-suppressive [39]. Moreover, the trend of miR-20a change in different kinds of cancer was variable. In addition, small sample sizes of pertinent studies lacking potent statistical power often lead to ambiguous conclusions. Two former meta-analyses suggested that high expression of miR-17~92 cluster is associated with unfavorable outcome of human cancers; however, authors did not specifically analyze miR-20a or made subgroup analysis based on tumor kinds or sample source, which undermine the credibility of these conclusions [20].

Circulating miRNAs were firstly identified in 2008 [40]. A vast majority of circulating miRNAs are derived from blood cells and endothelial cells and may act an important role in intercellular communication. During tumor progression, angiogenesis (formation of new vessels from preexisting vascular network) has been pathologically accelerated by multiple proangiogenic factors such as vascular growth factor (VEGF) and placenta growth factor (PIGF) [41]. Meanwhile, apoptotic and necrotic cells release miRNAs into the bloodstream and therefore some tissue-specific miRNAs are also detected in serum or plasma [42–44]. Circulating miRNAs are mainly capsulated in exosomes, cell-to-cell mediators of biological information, or binding to proteins, which makes these miRNAs highly stable in adverse physiological conditions [7, 45]. Moreover, compared with tissue miRNA, measuring plasma or serum miRNA is simple and injury-limited. However, it must be noted that perturbations of blood cells or hemolysis may greatly alter the level of circulating miRNAs [42] and therefore, blood-based phenomena should be taken into consideration when interpreting the results of circulating miRNAs.

Significant association between highly expressed circulating miR-20a and unfavorable OS/DFS of human cancers was identified in this study, which hinted that plasma/serum-based miR-20a acted as an oncogene. Previous studies have demonstrated overexpression of circulating miR-20a in a range of other malignant tumors such as colorectal cancer, cervical cancer, and hepatocellular carcinoma [46]. Circulating miR-20a could enhance tumor cell proliferation, migration, and invasion by targeting various pathways; for example, Du and his colleagues revealed that upregulated circulating miR-20a might suppress inhibitor *β* of nuclear factor- (NF-) *κ* B and therefore enhance activity of NF-*κ* B pathway and downstream molecules such as livin and survivin [47].

Unlike plasma/serum miR-20a, inconsistent results about the expression level and prognostic impaction of tissue-based miR-20a were reported in different kinds of tumors. In this investigation, a consistent tendency of tissue-based miR-20a expression change and survival was observed. Namely, for cancers with upregulated miR-20a level compared with paired nontumorous tissue, highly expressed miR-20a in tumor is associated with an unfavorable outcome, while for cancers with downregulated miR-20a level, high expression of miR-20a in tumorous tissue predicts a longer survival, which improves the conclusion of two former meta-analyses [19, 20]. MiR-20a could achieve diverse functions by targeting multiple mRNAs referred to as a targetome, and the cellular and genetic context may be decisive for the final effect of miRNAs [32, 39]. It is reported that upregulated miR-20a could promote colorectal cancer growth and progression by inhibiting multiple tumor-suppressive genes such as BIM and Smad4 [14, 48], while in breast cancer, miR-20a achieved inhibitory effect of cancer cell proliferation through directing targeting MAPK1/ERK2, a member of Ras/Raf/ERK pathway [32]. Impressively, existing evidences did not support aberrant circulating miR-20a in patients with breast cancer, cutaneous squamous cell carcinoma, hepatocellular carcinoma, oral squamous cell carcinoma, epithelial ovarian cancer, salivary adenoid cystic carcinoma, gallbladder carcinoma, and alveolar rhabdomyosarcoma compared with normal healthy controls [49]. Only in gastrointestinal tumors, upregulated miR-20a level in both serum/blood and tumorous tissue was confirmed and high expression of both circulating and tumorous miR-20a was associated with an unfavorable outcome.

These findings suggested miR-20a as a potential druggable target and provide an opportunity of using miR-20a mimics or miR-20a inhibitors (antimiR-20a) as innovative therapeutics for malignancies. With the advances of RNA biochemistry and delivery techniques, stable and effective miRNA-based agents have been put into clinical use [50, 51]. However, due to the complex interaction between receipt and donor cells and limited understanding of the underlying action mechanism and genomics of miR-20a in multiple cancer, clinical feasibility of the miR-20a as a therapeutic marker has a long way to go.

This investigation also revealed several directions of investigation for miR-20a. First, although multiple targeted genes and pathways of miR-20a have been identified in published studies, which one address key function in tumorigenesis and which factors resulted in the different change of miR-20a expression level in various cancers are undefined [9–11, 13–15, 17, 18, 34] (Tables 3 and 4). Second, some miRNAs (miR-17, miR-18a, miR-18b, miR-20b, miR-93, miR-106a, and miR-106b) are structurally homologous or expression/function-related to miR-20a [17, 52, 53]; polycistronic structures of these miRNAs may allow for reciprocal interactions. In the further, with the publication of more high-quality studies, meta-analyses with regard to other members of these miRNAs, especially miR-17 which was included in the same miRNA family with miR-20a [17, 53], may be conducted.

Some limitations of this meta-analysis merited consideration. First, inconsistent characteristics such as cut-off value and detection method may influence the outcomes. Second, many basic data were not provided in original studies. Third, although subgroup analyses were conducted, source of the heterogeneity was still not fully illustrated, indicating existence of negligible biased factors, i.e., surgical intervention, radiation, chemotherapy, mental state, and tumor characteristics. Fourth, only three electronic databases were searched. Although partial data also came from TCGA databases, some unpublished results may be missed. Last but not least, subgroup analysis, sensitivity analysis, and publication bias test were not available for some outcome measures due to the limited number of studies.

## 5. Conclusion

Elevated circulating miR-20a expression is significantly correlated with poor OS/DFS but the prognostic significance of tissue miR-20a has something to do with the trend of miR-20a change in different tumors. Highly expressed tissue-based miR-20a was associated with an unfavorable prognosis in cancers with upregulated miR-20a expression and favorable survival in malignancy with downregulated miR-20a expression. In a word, circulating and tissue-based miR-20a could serve as a reliable prognostic biomarker for patients with cancers. These results need additional validation for the limited number of studies.

## Figures and Tables

**Table 1:** Main characteristic of included studies about circulating miR-20a.

First author	Year	Country	Number of patients	Age, Median (range)	Cancer type	Stage range	Marker	Proposed target genes & pathways	Test method	Up- or down-regulated compared with healthy control	Number of high/low level of miR-20a	Cut-off value	Follow-up	Outcome	NOS score
Xu et al [23]	2018	China	196	59.5±10.31	NSCLC	I-III	Plasma miR-20a	BID, TRAIL	RT-PCR	Up-regulated	NR	the median value	56.7 months	OS, DFS	7
Yang et al [11]	2017	China	55	NR	gastric cancer	I-IV	Serum miR-20a	NFKBIB	microarray, qRT-PCR	Up-regulated	27/28	the median value	34 months	OS	7
Khare et al [24]	2017	Israel	25	18-85	lymphoma	I-IV	Plasma miR-20a	PTEN, pi3k/AKT	qRT-PCR	Up-regulated	NR	the median value	5 years	OS	6
Zhao et al [25]	2017	US	106	58	glioblastoma	NR	Plasma miR-20a-5p	TIMP-2	qRT-PCR	Up-regulated	NR	the median value	2 years	OS, DFS	7
Lin et al [9]	2014	Australia	97	68(46-87)	prostate cancer	NR	Plasma/serum miR-20a	RUNX1, CSF1R	qRT-PCR	Up-regulated	NR	NR	12 (3-62) months	OS	7
Sanfiorenzo et al [10]	2013	France	62	65.1	NSCLC	I-III	Plasma miR-20a	NR	microarray, qRT-PCR	Up-regulated	NR	NR	18months~	DFS	8
Wang et al [13]	2012	China	65	NR	gastric cancer	I-IV	Serum miR-20a	NR	qRT-PCR	Up-regulated	34/31	the median value	NR	OS	6

NR, not reported; NSCLC, non-small-cell lung cancer; NR, not reported; qRT-PCR, quantitative real time-polymerase chain reaction; OS, overall survival; DFS, disease-free survival.

**Table 2:** Main characteristic of included studies about tissue miR-20a.

First author	Year	Country	Number of patients	Age, Median (range)	Cancer type	Stage range	Marker	Proposed target genes & pathways	Test method	Up- or down-regulated compared with normal tissue	Number of high/low level of miR-20a	Cut-off value	Follow-up	Outcome	NOS score
Cheng et al [14]	2016	China	544	65	colorectal cancer	I~IV	miR-20a-5p	Smad4	qRT- PCR	Up-regulated	407/137	NR	6~12 years	OS, DFS	8
Chen et al [26]	2015	Asia/ Europe/ North America	580	67.9±13.2	colorectal cancer	I~IV	miR-20a-5p	BIM	qRT- PCR	Up-regulated	NR	NR	NR	OS	7
Mitani et al [17]	2013	US	64	NR	salivary adenoid cystic carcinoma	I~IV	micro-RNA-17-92	NR	qRT- PCR	Up-regulated	22/8	3	NR	OS	8
Chang et al [27]	2013	China	98	NR	gallbladder carcinoma	NR	miR-17/20a	Smad7	qRT-PCR	Up-regulated	71/27	NR	NR	OS	7
Zhang et al [28]	2013	China	138(training set)	NR	colon cancer	II	miR-20a-5p	NR	qRT- PCR	Up-regulated	NR	NR	66 months	DFS	7
			137(internal testing set)	NR	colon cancer	II	miR-20a-6p		qRT- PCR	Up-regulated	NR	NR		DFS	7
			460 (independent validation set)	NR	colon cancer	II	miR-20a-7p		qRT- PCR	Up-regulated	NR	NR		DFS	7
Valladares-Ayerbes et al [29]	2011	Spain	38	62.5(45-76)	gastrointestinal cancer	I~IV	miR-17; miR-20a; miR-21	E2F1, E2F2, E2F3, BIM, LRF,	qRT- PCR	Up-regulated	NR	NR	153 weeks (2-388 weeks)	OS, PFS	6
Reichek et al [30]	2011	US	123	NR	alveolar rhabdomyosarcoma	NR	micro-RNA-17-92	E2F	qRT- PCR	Up-regulated	NR	NR	NR	OS	6
Schetter et al [31]	2008	China	84;113	64.6(32-87); 55.8(32-84)	colon adenocarcinoma	I~IV	miR-20	NR	qRT- PCR	Up-regulated	NR	NR	NR	OS	7

Si et al [32]	2017	China	66 (cohort 1)	53.6	breast cancer	I~IV	miR-20	c-Myc	qRT- PCR	Down-regulated	30/36	NR	NR	OS	7
			40 (cohort 2)	56.1	breast cancer	I~IV	miR-21	c-Myc	qRT- PCR	Down-regulated	18/22	NR	NR	OS	7
			716 (cohort 3 from TCGA database)	59	breast cancer	I~IV	miR-22	c-Myc	qRT- PCR	Down-regulated	334/382	NR	NR	OS	7
Zhang et al [15]	2015	China	152	53.9	cutaneous squamous cell carcinoma	I-III	miR-20	NR	qRT- PCR	Down-regulated	54/98	NR	NR	OS	6
Fan et al [33]	2013	China	100	57.8/53.6	hepatocellular carcinoma	I-III	miR-20	Mcl-1	qRT- PCR	Down-regulated	50/50	NR	NR	OS, RFS	8
Chang et al [27]	2013	China	96	NR	oral squamous cell carcinoma	I~IV	miR-20	ITGb8	qRT- PCR	Down-regulated	34/33	NR	NR	OS	7
Marchini et al [34]	2011	Italy	144	9	epithelial ovarian cancer	I	miR-20	NR	qRT- PCR	Down-regulated	NR	NR	NR	OS, PFS	7

NR, not reported; TCGA, Tumor Cancer Genome Atlas; NR, not reported; qRT-PCR, quantitative real time-polymerase chain reaction; OS, overall survival; DFS, disease-free survival; PFS, progression-free survival; RFS, relapse-free survival.

**Table 3:** Meta-analyses of tumors with upregulated circulating or tissue miR-20a.

			Circulating miR-20a						Tissue miR-20a					
			No. of study	HR (95%CI)	*p*-value	I-square	*p*-value for heterogeneity	*p*-value of Egger' test	No. of study	HR (95% CI)	*p*-value	I-square	*p*-value for heterogeneity	*p*-value of Egger' test
OS	All	high vs low	6[9, 11, 13, 23–25]	1.71 (1.43 -2.04)	<0.01	3.3%	0.395	0.369	7[14, 17, 26, 27, 29–31]	2.74 (1.38-5.42)	<0.01	90.90%	<0.01	0.013
	Country	China (high vs low)	3[11, 13, 23]	1.94 (1.48-2.55)	<0.01	15%	0.308	/	5[17, 26, 29–31]	2.02 (1.14-3.58)	<0.01	77.60%	<0.01	0.001
		Other (high vs low)	3[9, 24, 25]	1.50 (1.18-1.91)	<0.01	0.00%	0.703	/	2[14, 27]	4.63 (1.48-14.50)	<0.01	84.20%	0.012	/
	Sample size	> 100 (high vs low)	2[23, 25]	2.13 (1.55-2.93)	<0.01	0.00%	0.409	/	3[26, 27, 30]	3.86 (1.28-11.63)	0.016	84.10%	<0.01	0.903
		< 100 (high vs low)	4[9, 11, 13, 24]	1.55 (1.27-1.91)	<0.01	0.00%	0.613	0.031	4[14, 17, 29, 31]	2.08 (1.04-4.19)	0.039	82.80%	<0.01	0.002
	Cancer type	gastrointestinal cancer (high vs low)	2[11, 13]	1.69 (1.22-2.34)	<0.01	0.00%	0.364	/	4[26, 27, 29, 31]	2.51 (0.99-6.39)	0.053	94.30%	<0.01	0.13
		others (high vs low)	4[9, 23–25]	1.74 (1.34-2.26)	<0.01	30.9%	0.227	0.702	3[14, 17, 30]	2.95 (1.74-5.00)	<0.01	0.00%	<0.01	0.806
	Calculation model of HR	Multivariate (high vs low)	5[9, 13, 23–25]	1.69 (1.39-2.04)	<0.01	11.0%	0.343	0.610	7[14, 17, 26, 27, 29–31]	2.74 (1.38-5.42)	<0.01	90.90%	<0.01	0.013
		Univariate (high vs low)	1[11]	2.32 (1.09-4.93)	/	/	/	/	/	/	/	/	/	/

DFS	All	high vs low	2[10, 23, 25]	1.90 (1.45-2.90)	<0.01	0.00%	0.688	/	4[14, 28]	2.68 (1.32-5.45)	<0.01	82.90%	<0.01	0.703
	Country	China (high vs low)	/	/	/	/	/	/	4[14, 28]	2.68 (1.32-5.45)	<0.01	82.90%	<0.01	0.703
		Other (high vs low)	/	/	/	/	/	/	/	/	/	/	/	/
	Sample size	> 100 (high vs low)	/	/	/	/	/	/	4[14, 28]	2.68 (1.32-5.45)	<0.01	82.90%	<0.01	0.703
		< 100 (high vs low)	/	/	/	/	/	/	/	/	/	/	/	/
	Cancer type	gastrointestinal cancer (high vs low)	/	/	/	/	/	/	4[14, 28]	2.68 (1.32-5.45)	<0.01	82.90%	<0.01	0.703
		others (high vs low)	/	/	/	/	/	/	/	/	/	/	/	/
	Calculation model of HR	Multivariate (high vs low)	/	/	/	/	/	/	4[14, 28]	2.68 (1.32-5.45)	<0.01	82.90%	<0.01	0.703
		Univariate (high vs low)	/	/	/	/	/	/	/	/	/	/	/	/

HR, hazard ratio; CI, confidence interval; OS, overall survival; DFS, disease free survival.

**Table 4:** Meta-analyses of tumors with downregulated tissue-based miR-20a.

Summary estimate	Subgroup analysis		No. of study	HR (95% CI)	*p*-value	I-square	*p*-value for heterogeneity	*p*-value of Egger' test
OS	All	low vs high	7[15, 18, 32–34]	3.48 (2.00-6.06)	<0.01	66.40%	<0.01	0.024
	Country	China (low vs high)	6[15, 18, 32, 33]	3.41 (1.80-6.47)	<0.01	68.40%	<0.01	0.04
		Other (low vs high)	1[34]	4.2 (1.99-8.87)	/	/	/	/
	Sample size	> 100 (low vs high)	4[15, 32–34]	2.70 (1.42-5.13)	<0.01	71.30%	0.015	0.253
		< 100 (low vs high)	3[18, 32]	5.21 (2.74-9.90)	<0.01	0.00%	0.462	0.129
	Cancer type	gastrointestinal cancer (low vs high)	/	/	/	/	/	/
		others (low vs high)	7[15, 18, 32–34]	3.48 (2.00-6.06)	<0.01	66.40%	<0.01	0.024
	Calculation model of HR	Multivariate (low vs high)	6[15, 32–34]	3.41 (1.80-6.47)	<0.01	68.40%	<0.01	0.04
		Univariate (low vs high)	1[18]	4.2 (1.99-8.87)	/	/	/	/

PFS & RFS	All	low vs high	2[33, 34]	4.05 (2.89-5.66)	<0.01	0.00%	0.345	/
	Country	China (low vs high)	1[33]	4.28 (3.32-6.74)	/	/	/	/
		Other (low vs high)	1[34]	2.55 (0.93-7.04)	/	/	/	/
	Sample size	> 100 (low vs high)	2[33, 34]	4.05 (2.89-5.66)	<0.01	0.00%	0.345	/
		< 100 (low vs high)	/	/	/	/	/	/
	Cancer type	gastrointestinal cancer (low vs high)	/	/	/	/	/	/
		others (low vs high)	2[33, 34]	4.05 (2.89-5.66)	<0.01	0.00%	0.345	/
	Calculation model of HR	Multivariate (low vs high)	2[33, 34]	4.05 (2.89-5.66)	<0.01	0.00%	0.345	/
		Univariate (low vs high)	/	/	/	/	/	/

HR, hazard ratio; CI, confidence interval; OS, overall survival; PFS, progression free survival; RFS, relapse free survival.

## Supplementary Materials

Supplementary Material The PRISMA Checklist for this systematic review and meta-analysis describing the page which every item located in.
